# ECG Images dataset of Cardiac and COVID-19 Patients

**DOI:** 10.1016/j.dib.2021.106762

**Published:** 2021-01-18

**Authors:** Ali Haider Khan, Muzammil Hussain, Muhammad Kamran Malik

**Affiliations:** a Department of Computer Science, School of System & Technology, University of Management and Technology Lahore, Pakistan; b Department of Computer Science, University of the Punjab Lahore, Pakistan

**Keywords:** Cardiac care, ECG dataset, COVID-19, Health care institutes, Deep learning

## Abstract

The study contains the dataset of ECG images of Cardiac and COVID-19 patients. This rare dataset contains 1937 distinct patient records, data is collected using ECG Device ‘EDAN SERIES-3’ installed in Cardiac Care and Isolation Units of different health care institutes across Pakistan. The collected ECG images data were manually reviewed by medical professors using Telehealth ECG diagnostic system, under the supervision of senior medical professionals with experience in ECG interpretation. The manual reviewing process of ECG images took several months to review the five distinct categories (COVID-19, Abnormal Heartbeat, Myocardial Infarction (MI), Previous History of MI, and Normal Person). The collected data contains 12 leads-based ECG images dataset can be used by Data Scientist, IT Professional and Medical Research Institutes to design, compare, fine-tune classical techniques and Deep learning methods in studies focused on COVID-19, Arrhythmia, and other cardiovascular conditions. The dataset contains rare categories of patients that may be used for the development of automatic diagnosis tool for healthcare institutes.

## Specifications Table

**Table:** 

Subject	Computer Science, Health and Medical Sciences, Medical Imaging
Specific subject area	Medical Imaging Analysis
Type of data	Image
How data were acquired	Data is collected using ECG Device ‘EDAN SERIES-3’ installed in Cardiac Care and Isolation Units of different health care institutes across Pakistan.
Data format	RAW
Parameters for data collection	12 Leads based ECG Images Data is collected from EDAN SERIES - 3 devices of 500 Hz sampling rate.
Description of data collection	ECG images were collected from different health care institutes across Pakistan. All collected data were manually reviewed by team of Senior Medical Professionals to remove all ambiguous and misleading images from collected data. Telehealth ECG diagnostic system is used in the reviewing process.
Data source location	Institution: Ch. Pervaiz Elahi Institute of Cardiology City/Town/Region: Multan/Punjab Country: Pakistan Latitude and longitude: 30.1920° N, 71.4505° E Institution: Nishtar Medical University City/Town/Region: Multan/Punjab Country: Pakistan Latitude and longitude: 30.2033° N, 71.4412° E Institution: Punjab Institute of Cardiology City/Town/Region: Lahore/Punjab Country: Pakistan Latitude and longitude: 31.5382° N, 74.3362° E
Data accessibility	Repository name: Mendeley Data Data identification number: 10.17632/gwbz3fsgp8.1 Direct URL to data: http://dx.doi.org/10.17632/gwbz3fsgp8.1

## Value of the Data

• The data is important for screening the insight of Cardiac and COVID-19 patients and their relationships.
• 12 lead ECG images dataset can be used by Data Scientist, IT Professional, and Medical Research Institutes to design, compare, fine-tune, classical techniques and Deep learning methods in studies focused on COVID-19, Arrhythmia, and other cardiovascular conditions.
• This dataset contains rare categories of patients that may be used for the development of automatic diagnosis tool for healthcare institutes.

## 1 Data Description

12-lead based standard ECG images collected from distinct patients from diverse cardiac institutes across Pakistan. The ECG images do not contain any personal information about the patient. All ECG images have been annotated by several medical experts [5]. Below Table 1 reports the number of images for the different cases.

**Table 1:** ECG Dataset Detail.

Sr.	Category / Folder Name	No. of Distinct ECG Images	Sample Rate	Leads
1	COVID-19 Patients	250	500 Hz	12 – Leads
2	Normal Person ECG Images	859		
3	Myocardial Infarction Patient	77		
4	Patients with Previous History of Myocardial Infarction	203		
5	Patients with Abnormal Heartbeat	548		

### 1.1 COVID-19

Coronavirus commonly known as COVID -19 virus was emerged in late December 2019 in the city of China and expanded globally [1]. WHO declared a public health emergency in January 2020 due to the high spread of this virus [2]. This is an infection mainly caused by a touching or interconnection with an infected person and most people infected by the COVID-19 virus will experience shortness of breath and respiratory illness and maybe recovered with or without special treatment [1].

### 1.2 Normal Person

A normal person in medical terms is a person that acts or functioning naturally or lacks any observable abnormalities or any kind of deficiencies [3].

### 1.3 Myocardial Infarction

Myocardial infarction (MI) commonly known as a heart attack, occurs when the flow of blood decreases or stops to a part of the heart, causing severe damage to the heart. Most common symptom is chest pain or discomfort which may travel into the shoulder, arm, back, neck, or jaw. MI is a type of acute coronary syndrome, which describes a sudden or short-term change in symptoms related to blood flow to the heart and it can be detected by Electrocardiogram (ECG) sensing for proper diagnosis of the patient [4].

### 1.4 Previous History of Myocardial Infarction

Patients that are recently recovered from Myocardial Infarction (MI) or Heart Attack.

### 1.5 Abnormal Heartbeat

ECG images of the Patients that are suffering from Abnormal Heartbeat recently recovered from COVID-19 and Myocardial Infarction and have symptoms of shortness of breath or respiratory illness.

## 2 Experimental Design, Materials and Methods

### 2.1 Data Collection Design

**Fig. 1:**
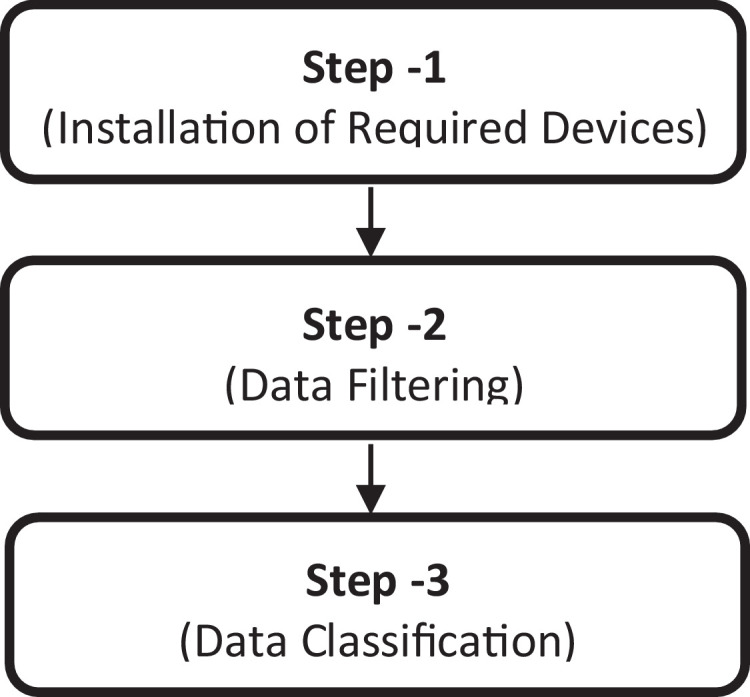
Data Collection Steps.

In this study, the authors created an ECG image dataset from distinct patients with a confirmed diagnosis of COVID-19 and Cardiac diseases who have been treated in healthcare institutes. EDAN SERIES-3 devices were installed for data collection and the telehealth diagnostic assistant tool was utilized by the authors to consult the collected images from the health care professionals. Below Fig. 1 shows the essential steps required for data collection.

### 2.2 Data Filtering Process

EDAN ECG Devices were installed for data collection. The collected data was reviewed in four-step process.

#### 2.2.1 ECG Device Configuration

All ECG devices used for data gathering were configured as ‘ON’ for critical alerts. The ECG technicians were trained to respond to all types of notifications/alerts of EDAN ECG devices so that ECG technicians can perform all precautionary measures while performing ECG. This step is important for capturing ECG images more accurately.

#### 2.2.2 Data Privacy

All personal information of the patient was removed from collected ECG images.

#### 2.2.3 Data Annotation

Collected ECG Images were reviewed by several medical professors using Telehealth ECG diagnostic system, under the supervision of senior medical professionals with experience in ECG interpretation. The manual reviewing process of ECG images took several months to review the five distinct categories (COVID-19, Abnormal Heartbeat, Myocardial Infarction (MI), Previous History of MI, and Normal Person).

#### 2.2.4 Data Validation

All Collected Data was finally reviewed by team of Senior Medical Professionals to remove all ambiguous and misleading images from the selected data.

### 2.3 Data Classification

Collected ECG images are classified into five distinct groups. i) ECG images of the COVID-19 Patients were collected from the “COVID-19 isolation unit”. ii) Normal group ECG images were collected from “Patient's attendants and visitors”. iii) ECG images of the Myocardial Patients were collected from the “Cardiac Care Unit”. iv) ECG images of the Patients recently recovered from Myocardial Infarction were collected from the “Out-Patient Department”. v) ECG images of the Patients suffering from Abnormal Heartbeat who recently recovered from COVID-19 and Myocardial Infarction that have symptoms of shortness of breath collected from the “Out-Patient Department”.

## Ethics Statement

All ECG Images of the dataset are fully anonymized and all personal information was removed and this dataset is permits use, sharing, adaptation, distribution, and reproduction in any medium or format as long as you give appropriate credit to the original author(s). All protocols were approved by Medical Superintended CPEIC Multan Pakistan wide Office Order No. 8607 on dated 29-04-2020.

## CRediT Author Statement

**Ali Haider Khan:** Conceptualization, Data curation, Writing- Original draft preparation; **Muzammil Hussain**: Writing- Reviewing and Editing, Project administration, Supervision; **Muhammad Kamran Malik**: Supervision.

## Declaration of Competing Interest

The authors declare that they have no known competing financial interests or personal relationships which have or could be perceived to have influenced the work reported in this article.
